# The effects of optical, electrical and chemical stimulation on serotonin release from median raphe and hippocampus of mice

**DOI:** 10.1186/2193-1801-4-S1-P14

**Published:** 2015-06-12

**Authors:** Flora Goloncser, Romeo Ando, Dora Zelena, Jozsef Haller, Beata Sperlagh

**Affiliations:** Institute of Experimental Medicine of the Hungarian Academy of Science, Hungary; Semmelweis University School of PhD Studies, Hungary

**Keywords:** channelrhodopsin-2, median raphe nucleus, serotonin

The present study has examined several characteristics of the release of [3H]5-HT from the median raphe nucleus (MRN) and hippocampus in terms of its dependence of nerve impulse. We used electrical stimulation and the sodium channel opener veratridine, which excite all of the neuronal processes in the stimulation field, and optogenetics to selectively stimulate those terminals which express channelrhodopsin-2 (ChR2) and compared 5-HT release evoked by electrical and chemical depolarization and by light. We injected an adeno-associated virus containing DNA construct encoding ChR2 into the MRN of mice and investigated [3H]5-HT release from MRN and hippocampal slices. Serotonergic nerve terminals was locally stimulated with 473 nm light (blue laser diode) and electrically by bipolar electrode and by veratridine and transmitter release was monitored by collecting the effluent in a fraction collector. Electrical filed stimulation and veratridine resulted in a significantly increase in the efflux of 5-HT, whereas optical stimulation of ChR2 expressing nerve terminals at various frequencies (10, 20, 50, 100 Hz) elicited only a negligible increase in 5-HT release either from the hippocampus or from the MRN itself. The electrically induced release of radioactive neurotransmitter was completely inhibited by perfusion with tetrodotoxin. We have also applied the 5-HT transporter inhibitor, fluoxetine and the GABAA blocker bicuculline to relieve released 5-HT from re-uptake and any endogenous inhibition. Nevertheless, the effect of optical stimulation remained closed to the detection limit under these condition. In conclusion, whereas our method is suitable to detect [3H]5-HT efflux in response to ongoing neuronal sodium channel activity its sensitivity is too low to detect transmitter efflux evoked by focal optogenetic stimulation. The most likely reason for the failure of detection of 5-HT efflux is that ChR2 is expressed only by a small subpopulation of nerve terminals.

